# Vedolizumab treatment in adults with immunodeficiency-associated enteropathy: a case series

**DOI:** 10.3389/fimmu.2026.1953966

**Published:** 2026-10-05

**Authors:** Ernestina Angarola, María Josefina Etchevers, Juan Andres De Paula, Maria Florencia Sanchez, Juan Pablo Santino, Diana Liberatore

**Affiliations:** 1 Immunology Section, Hospital Italiano de Buenos Aires, Buenos Aires, Argentina; 2 Internal Medicine Department, Hospital Italiano de Buenos Aires, Buenos Aires, Argentina; 3 Gastroenterology Section, Hospital Italiano de Buenos Aires, Buenos Aires, Argentina; 4 Pathology Department, Hospital Italiano de Buenos Aires, Buenos Aires, Argentina

**Keywords:** combined immunodeficiency, common variable immunodeficiency, enteropathy, inborn errors of immunity, villous atrophy

## Abstract

**Background:**

Inflammatory gastrointestinal disease is a common manifestation of inborn errors of immunity (IEI) and may affect any segment of the gastrointestinal tract. Vedolizumab, a gut-selective anti-integrin approved for inflammatory bowel disease, has been scarcely studied in IEI. We describe our experience with vedolizumab in adults with immunodeficiency-associated enteropathy.

**Methods:**

We conducted a retrospective case series of adults with humoral or combined immunodeficiency and inflammatory gastrointestinal disease treated with vedolizumab at a single tertiary referral center. Clinical, endoscopic, laboratory and histopathological responses were assessed.

**Results:**

Four adult patients with immunodeficiency-associated enteropathy received vedolizumab. Two patients achieved sustained clinical and endoscopic/histological remission, both of whom had predominant villous atrophy (one with TRAF3 haploinsufficiency, and the other had multiple autoimmune manifestations, refractory immune thrombocytopenia and agammaglobulinemia following rituximab). Another patient with combined immunodeficiency and protein-losing enteropathy had marked clinical improvement with mild histologic response, but relapsed at two years in the setting of norovirus and Clostridium difficile infection. In contrast, a woman with common variable immunodeficiency, autoimmune hepatitis, and severe refractory pancolitis failed to respond to vedolizumab and subsequently developed CMV superinfection, requiring treatment escalation.

**Conclusions:**

In this case series, vedolizumab was associated with clinical improvement predominantly in patients with villous atrophy rather than refractory colitis. These findings are hypothesis-generating and support further evaluation of vedolizumab as a therapeutic option for selected patients with immunodeficiency-associated enteropathy.

## Introduction

Inborn errors of immunity (IEIs), also known as primary immunodeficiencies, comprise more than 550 genetically defined entities affecting humoral, cellular, combined, and innate immune function (1). Immune dysregulation leading to symptomatic gastrointestinal disorders affects 5% to 50% of patients with IEI, including celiac disease-like, lymphocytic gastritis, granulomatous disease, microscopic colitis, inflammatory bowel disease (IBD)-like and lymphoma (2).

Among patients with IEI and gastrointestinal involvement requiring endoscopic evaluation, immune dysregulation disorders - particularly CTLA4 deficiency, X-linked lymphoproliferative syndrome, and IPEX syndrome- and phagocyte defects, predominantly chronic granulomatous disease, are the most frequently represented categories, followed by predominantly antibody deficiencies (3). More than 80 monogenic causes of IBD have now been identified, the majority overlapping with IEI; among these, interleukin-10 (IL-10) and IL-10 receptor signaling defects represent the most striking example of life-threatening, infantile-onset IBD (4). Gastrointestinal manifestations in IEI are often debilitating, notoriously difficult to treat and contribute to increased morbidity and mortality (5).

The infectious risk associated with conventional systemic immunosuppressants for inflammatory gastrointestinal disorders is of significant concern in patients with IEI. Vedolizumab, a humanized monoclonal antibody against the α4β7 integrin that inhibits lymphocyte migration into intestinal tissue, was approved for the treatment of adults with moderate to severe IBD in 2014 (6). Unlike systemic immunosuppressants, its mechanism of action is restricted to gut-homing lymphocytes, sparing systemic immune surveillance. The gut-selective mechanism of action has not been linked to an increased risk of opportunistic infections, positioning this medication as a potentially beneficial option for patients with immunodeficiencies (7). However, there is insufficient evidence about the utility of vedolizumab in patients with IEI. Some authors reported benefits treating gut inflammation in common variable immunodeficiency (CVID) patients, whereas others did not find it helpful (5, 7, 8). Also, there are limited data regarding the utility of vedolizumab in other IEI.

## Methods

We conducted a retrospective, descriptive case series at a single tertiary care center, Hospital Italiano de Buenos Aires, Argentina. We reviewed the Institutional Registry of Rare Diseases. Patients were included in the IEI section if immunological and/or genetic evaluation fulfilled the IUIS criteria for an IEI (1). Patients with a secondary immunodeficiency followed in our section were excluded. Nevertheless, we opted to include one remarkable patient (Case 4) who received rituximab but has a high clinical suspicion of an underlying IEI that could not yet be genetically confirmed. We included patients aged 18 years or older with humoral or combined immunodeficiency and inflammatory gastrointestinal disorders who received treatment with vedolizumab between January 2020 and June 2026. Vedolizumab was administered intravenously at a standard dose of 300 mg at weeks 0, 2 and 6, followed by maintenance infusions every 8 weeks.

Clinical response was defined as improvement in stool frequency/consistency, weight gain, and/or discontinuation or reduction of corticosteroid dose. Endoscopic response was defined as resolution or improvement of mucosal lesions compared with baseline. Histopathological response was defined as reduction in inflammatory infiltrate or improvement of villous architecture on consecutive biopsies. Laboratory response was additionally assessed when available, and was defined as an increase in serum IgG (without changes in doses) and/or albumin levels, and/or a decrease in fecal calprotectin and/or alpha-1 antitrypsin clearance, compared with pretreatment values. As this is a retrospective case series, these parameters were not obtained at pre-specified time points but were recorded whenever available in the clinical records, and are summarized for every patient in **Table 1**.

**Table 1:** Clinical, laboratory and endoscopic/histopathological findings before and after treatment with vedolizumab.

Case	Findings before treatment with vedolizumab	Follow-up 1	Follow-up 2	Follow-up 3	Overall response
Case 1	Weight: 67 kg Stool frequency: 3–4 watery/day Use of steroids: prednisone 6 mg Hospitalization: No PN: No Albumin^+^: 3.5 g/dL IgG levels: 539 mg/dL Calprotectin*: 2065 µg/g Duodenum: Marsh IIIc, active duodenitis Ileum: follicular hyperplasia, chronic ileitis Colon: chronic active colitis	~1 year Weight: 69 kg Stool frequency: 1/day normal Use of steroids:add prednisone 10 mg Hospitalization: No PN: No Albumin:3.6 g/dL IgG levels: 692 mg/dL Calprotectin:838 µg/g Duodenum: villous architecture restored; active duodenitis persists Ileum: follicular hyperplasia Colon: focal active colitis in rectum	~2 years Weight: 75 kg Stool frequency: 1/day normal Use of steroids: No Hospitalization: No PN: No Albumin: 4.08 g/dL IgG levels: 989 mg/dL Calprotectin: 225 µg/g Duodenum: villous architecture restored; peptic ulcer		Clinical remission Histological/endoscopic response
Case 2	Weight: 41 kg Stool frequency:3-4/day Use of steroids: 9 mg budesonide Hospitalization: Yes PN: Yes Albumin: 3.9 g/dL IgG levels: 134 mg/dL Alpha-1-antitrypsin clearance: 422 mL/24 h Calprotectin: 187 µg/g Duodenum: Marsh IIIc, intraepithelial lymphocytosis >25%, absent plasma cells	~1 year: Weight: 44.5 kg Stool frequency: 1/day Use of steroids: 3 mg budesonide Hospitalization: No PN: No Albumin: 4.2 g/dL IgG levels: 412 mg/dL Alpha-1-antitrypsin clearance: 18.7 mL/24 h (normalized) - Duodenum: chronic atrophic duodenitis Marsh IIIb, intraepithelial lymphocytes, single non-necrotizing granuloma	~2 years: Weight: 41.8 kg Stool frequency: 4-5/day Steroids: 6 mg budesonide Hospitalization: Yes PN: No Albumin: 3.4 g/dL IgG levels: 227 mg/dL Alpha-1-antitrypsin clearance: 132 mL/24 h - Duodenum: persistent villous atrophy Marsh IIIb, intraepithelial lymphocytes >25%		Initial clinical response, mild histologic response. Subsequent clinical relapse after two years of treatment, norovirus and *C.difficile*
Case 3	Weight: 64 kg Stool frequency: 9–10 watery/day Use of steroids: 9 mg budesonide Hospitalization: Yes PN: Yes Albumin^+^: 3.6 g/dL IgG levels: 743 mg/dL Calprotectin: 371 µg/g Duodenum: normal Ileum: follicular hyperplasia, active ileitis Colon: severe active chronic colitis, absent plasma cells	After 4 doses: Weight: 64 kg Stool frequency: 6-10/day Use of steroids: 9 mg budesonide Hospitalization: Yes PN: No Albumin: 3.2 g/dL IgG levels: 553 mg/dL Colon: severe active colitis with CMV viral inclusions	—		No response; CMV superinfection
Case 4	Weight: 52 kg Stool frequency: 3–4 watery/day Use of steroids: 9 mg budesonide Hospitalization: No PN: No Albumin^+^: 3.1 g/dL IgG levels: 610 mg/dL Calprotectin: 332 µg/g Duodenum: Marsh IIIc, intraepithelial lymphocytosis >30%, absent plasma cells Ileum: follicular hyperplasia Colon: no inflammatory activity	~1 year: Weight: 59 kg Stool frequency: 1/day Use of steroids: No Hospitalization: No PN: No Albumin: 3.1 g/dL IgG levels: 717 mg/dL Calprotectin: 794 µg/g Duodenum: restored villous architecture, normal intraepithelial lymphocyte count, absent plasma cells	~2 years: Weight: 62 kg Stool frequency: 1/day Use of steroids: add 6 mg budesonide 2 months Hospitalization: No PN: No Albumin: 4.3 g/dL IgG levels: 806 mg/dL Calprotectin: 203 µg/g Duodenum: partial villous atrophy	~4 years: Weight: 65 kg Stool frequency: 1/day Use of steroids: No Hospitalization: No PN: No Albumin: 4.5 g/dL IgG levels: 1049 mg/dL Calprotectin: 48 µg/g Duodenum: restored villous architecture Ileum and colon: no inflammatory activity	Sustained clinical and histologic remission with one mild histologic relapse resolved with short-course of oral budesonide, in remission for two years since then

^+^ Albumin normal range: 3.2–5 g/dL *Calprotectin normal range: 5–45 ug/g ^#^Alpha-1-antitrypsin clearance normal range: <20 mL/24 h. PN, parenteral nutrition.

Prior to initiation of biologic therapy, all patients underwent evaluation by an infectious disease specialist to rule out active gastrointestinal infection, including stool cultures and PCR testing, as well as histopathological assessment of tissue samples for cytopathic effect complemented by PCR-based testing for viral pathogens such as cytomegalovirus (CMV).

This Institutional Registry of Rare Disease was approved by the Ethics Committee (Project number 7014) at Hospital Italiano de Buenos Aires. Written informed consent for research purposes is obtained before starting follow-up at this institution and also to be included in the registry.

## Results

A total of 105 adult patients across all IUIS categories were identified (86 with predominantly antibody deficiencies, 8 with combined immunodeficiency, and 11 with other IUIS categories), of whom 12 (11%) had inflammatory gastrointestinal disease, all within the subgroup with predominantly antibody or combined immunodeficiency (12/94, 12.8%). Four received vedolizumab at some point during follow-up. We describe the clinical features of gastrointestinal involvement and treatment response in this subgroup.

### Case 1

A 38-year-old man with combined immunodeficiency with immune dysregulation had a longstanding history of enteropathy with chronic diarrhea with 3–4 watery stools per day. beginning at the age of 14 years, with duodenal, ileal, and colonic involvement requiring recurrent topical and systemic corticosteroid therapy. By the age of 29 years, biopsies demonstrated complete villous atrophy with an inflammatory infiltrate in the lamina propria and increased eosinophils and chronic proctocolitis with foci of neutrophilic exocytosis.

He also experienced recurrent respiratory tract infections, prompting his first immunological evaluation in 2020 at the age of 33 years. The finding of hypogammaglobulinemia, impaired response to both protein and polysaccharide vaccines, and CD4+ lymphopenia established the diagnosis of combined immunodeficiency. Immunoglobulin replacement therapy and antibiotic prophylaxis were initiated, resulting in no further infections. He continued to experience persistent diarrhea, and repeat endoscopic evaluation showed persistent complete villous atrophy, with active ileitis and colitis. Although corticosteroids achieved clinical improvement, he became steroid-dependent. Vedolizumab was therefore initiated in 2021, achieving good symptomatic control with successful steroid withdrawal and normalization of stool frequency. After one year of treatment, mild endoscopic activity was noted only in the rectum, which resolved after the addition of low-dose prednisone for 2 months. He was later able to discontinue corticosteroids and remained asymptomatic on vedolizumab for three years, with a weight gain of 8 kg over this period. IgG levels also increased, likely reflecting improved control of enteropathy.

In 2023, a genetic diagnosis was established with the identification of a *de novo* pathogenic *TRAF3* variant resulting in haploinsufficiency. In 2024, he developed polyarthritis, requiring the addition of tofacitinib, and vedolizumab was discontinued. Tofacitinib provided good control of both the articular and gastrointestinal manifestations. He subsequently self-discontinued tofacitinib for one year, during which villous atrophy, ileitis, and colitis relapsed. Reintroduction of tofacitinib resulted in a favorable clinical response.

### Case 2

A 24-year-old man with combined immunodeficiency had a history of recurrent pneumonia since the age of one year, as well as short stature with delayed bone age. At the age of four years he was diagnosed with combined immunodeficiency and started immunoglobulin replacement therapy and antibiotic prophylaxis. At the age of 16 years, he was diagnosed with high-risk stage IVB Hodgkin lymphoma with pulmonary involvement and bulky mediastinal disease, for which he received ESHAP chemotherapy followed by radiotherapy. A heterozygous variant of uncertain significance in *TNFRSF13B* (*TACI*) was identified. As this finding did not fully explain the patient’s phenotype, whole-exome sequencing remains under periodic reanalysis.

At the age of 20 years, he developed autoimmune enteropathy characterized by chronic diarrhea and severe nutritional compromise, requiring hospitalization with temporary parenteral nutrition and enteral nutritional support. Endoscopic biopsies demonstrated complete villous atrophy, and a markedly elevated fecal alpha-1 antitrypsin clearance (422 mL/24 h; normal <20 mL/24 h). Despite corticosteroid therapy, clinical response was poor.

Vedolizumab was subsequently initiated, resulting in normalization of stool frequency, weight gain, discontinuation of parenteral nutrition, and normalization of alpha-1 antitrypsin clearance to 18.7 mL/24 h. One year later, follow-up upper endoscopy showed mild endoscopic and histologic response, with residual chronic atrophic duodenitis, and although he had better symptomatic control, he was not able to discontinue budesonide. Two years after initiating vedolizumab, he experienced worsening diarrhea. Repeat endoscopy revealed chronic duodenitis with inflammatory activity, complete villous atrophy, and recurrent elevation of alpha-1 antitrypsin clearance. He required rehospitalization, during which norovirus and *Clostridium difficile* infection were identified. His gastrointestinal symptoms improved following treatment with oral vancomycin. At present, escalation to an alternative therapeutic regimen is being considered.

### Case 3

A 51-year-old woman with common variable immunodeficiency (CVID) and autoimmune hepatitis, diagnosed at the age of 38 years. She developed chronic diarrhea at the age of 42 years and an initial endoscopic evaluation demonstrated active proctocolitis, with partial clinical improvement following treatment with topical and systemic corticosteroids and mesalamine. Subsequent endoscopic examinations revealed ileitis in addition to persistent proctocolitis. Histopathological analysis showed chronic proctocolitis with foci of acute inflammatory activity, including cryptitis, crypt abscesses, and a mixed inflammatory infiltrate in the lamina propria.

Azathioprine was initiated, resulting in a favorable clinical response, but was discontinued due to severe leukopenia. She subsequently developed progressively worsening bloody diarrhea, with up to 10 stools per day, requiring hospitalization. During hospitalization, following an infectious disease evaluation that ruled out active infection, infliximab was initiated; however, no significant clinical benefit was observed. By that time, endoscopic and histologic findings had progressed to severe active chronic pancolitis. At 48 years of age, she received four doses of vedolizumab without clinical improvement and subsequently developed extensive CMV-associated colitis.

Treatment was then switched to ustekinumab. Although endoscopic and laboratory findings continue to demonstrate ongoing inflammatory activity, she has shown gradual clinical improvement, with weight gain, reduced diarrhea (although not complete resolution) and healing of the previously observed deep colonic ulcers. She continues to require a low dose of prednisone.

### Case 4

A 53-year-old woman was diagnosed with alopecia areata at the age of 16, which progressed to alopecia universalis at 45, and with vitiligo and Hashimoto thyroiditis at the age of 26. At the same age, she developed refractory immune thrombocytopenia requiring rituximab and splenectomy, with subsequent agammaglobulinemia.

Also at the age of 26, she was diagnosed with celiac disease based on characteristic histological findings in endoscopic duodenal biopsies, despite negative celiac-specific antibodies. At the age of 45, her diarrhea worsened; upper gastrointestinal endoscopy and capsule endoscopy revealed complete villous atrophy extending from the duodenal bulb to the proximal ileum, refractory to corticosteroid therapy. Azathioprine was initiated but discontinued due to severe leukopenia. During this period, she developed pulmonary CMV infection and severe COVID-19.

At 47 years of age, considering persistent diarrhea and severe infections, vedolizumab was initiated. After five doses, she achieved both clinical and endoscopic improvement. Vedolizumab was subsequently discontinued, and she remained free of villous atrophy for two years. Thereafter, partial villous atrophy recurred and improved after a two-month course of budesonide. Her most recent endoscopic evaluation, performed in 2026, demonstrated complete resolution of villous atrophy with no evidence of ongoing mucosal inflammation.

Given the high suspicion of an underlying IEI, a targeted genetic panel was performed, which did not identify any pathogenic variants. Although she received rituximab, persistent agammaglobulinemia 17 years after her last dose, together with a broad dysregulation phenotype, is highly suggestive of an underlying primary IEI rather than a purely rituximab-induced secondary hypogammaglobulinemia; whole-exome sequencing is pending.

Clinical and immunological features in patients treated with vedolizumab are shown in **Table 2** and the main endoscopic and biopsy findings before and after treatment are summarized in **Table 1**. Representative histopathological features are shown in **Figure 1**.

**Table 2:** Clinical and immunological features in patients treated with vedolizumab.

Clinical and immunological features	Case 1	Case 2	Case 3	Case 4
Age of first symptoms related to IEI (years)	14	1	38	16
Age of first gastrointestinal symptoms (years)	14	20	42	26
Age of immunodeficiency diagnosis (years)	33	4	38	30
Extraintestinal clinical features	Recurrent RTI, warts, penile intraepithelial neoplasia, splenomegaly, polyarthritis	Recurrent RTI, bronchiectasis, HSM, portal hypertension, Hodgkin lymphoma, short stature with delayed bone age	Autoimmune hepatitis, esophageal candidiasis, recurrent RTI	ITP, splenectomy, thyroiditis, vitiligo, alopecia universalis, RTI, CMV pneumonia, severe COVID-19
Immunoglobulin levels at diagnosis IgG (800–1700 mg/dL) IgA (70–400 mg/dL) IgM (50–300 mg/dL) IgE (0.1–100 UI/mL)	539 48 40 2	<200 <4 8 -	370 36 19 <0.1	<200 <4 <10 <0.1
Polysaccharide response	Absent	Decreasing titers	Poor response	Absent
Lymphocyte subsets at diagnosis (cells/uL) CD19+(110–570 cells/uL) CD4+(530–1300 cells/uL) CD8+ (330–920 cells/uL) CD56+ (70–597 cells/uL)	515 (22%) 196 (28%) 413 (59%) 70 (3%)	8 (0.9%) 123 (14%) 687 (86%) 75 (8.4%)	3 (0.3%) 281 (36%) 337 (43%) 181 (21%)	1 (1%) 358 (42%) 275 (32%) 221 (26%)
Genetic findings	*TRAF3* c.1219 C>T heterozygous	*TNFRSF13B* c.171G>C (heterozygous, VUS)	Not performed due to limited access	Panel negative, WES pending results
Immunoglobulin replacement	IV, 400 mg/kg	IV, 800 mg/kg	SC, 160 mg/kg/week	SC, 125 mg/kg/week
Previous treatment for gastrointestinal disease	Topical and systemic corticosteroids	Topical and systemic corticosteroids	Topical and systemic corticosteroids, azathioprine, infliximab	Topical and systemic corticosteroids, azathioprine
Infections during vedolizumab treatment	None	Norovirus, *Clostridium difficile*	CMV	None

CMV, cytomegalovirus; ITP, immune thrombocytopenic purpura; IV, intravenous; HSM, hepatosplenomegaly; RTI, respiratory tract infection; SC, subcutaneous; VUS, variant of uncertain significance; WES, whole-exome sequencing.

**Figure 1:**
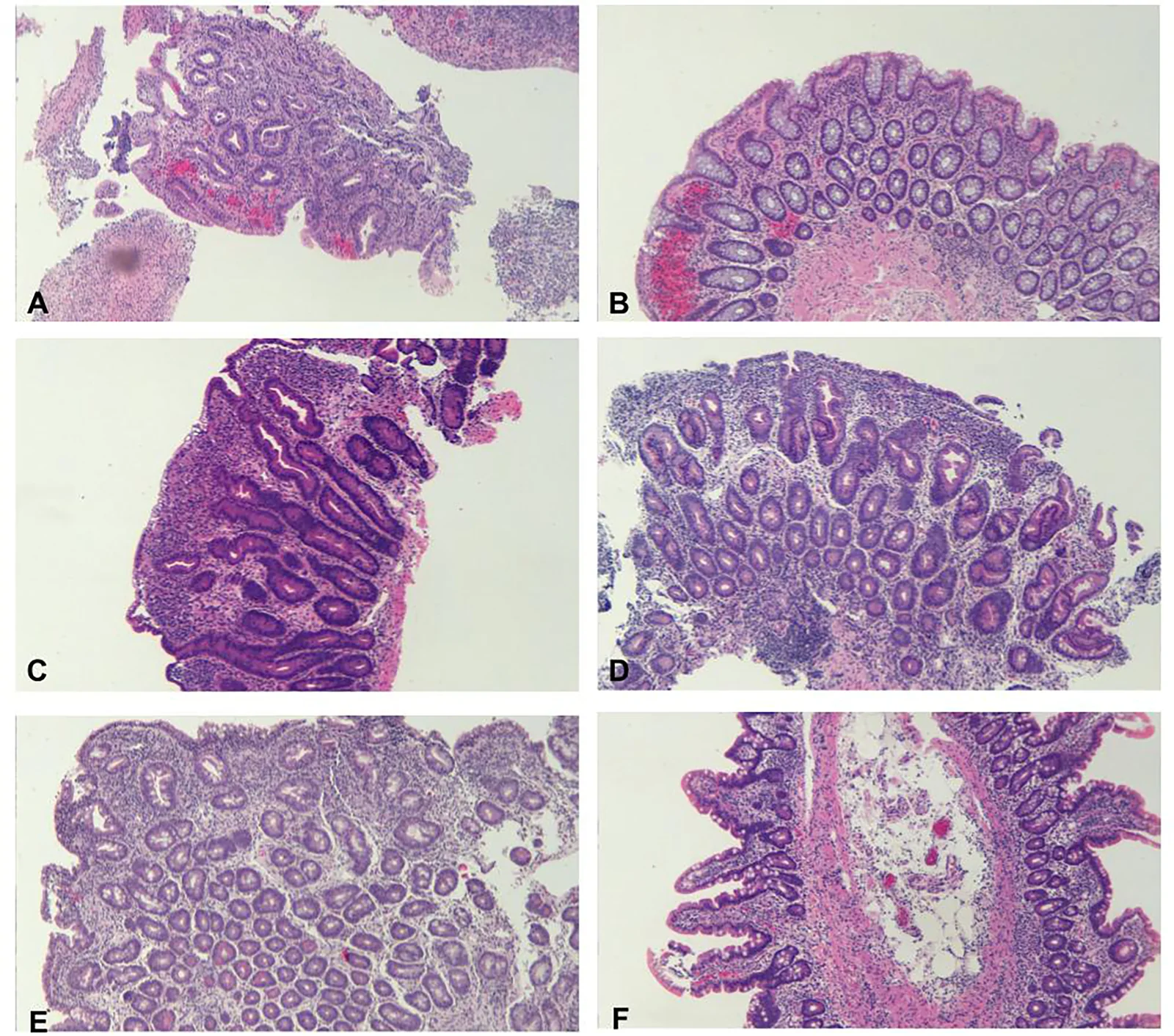
Histological features of duodenum before and after vedolizumab treatment. Hematoxylin and eosin staining, 100× magnification. **(A, B)** Case 1: complete villous atrophy before vedolizumab treatment **(A)** and restoration of normal villous architecture after treatment **(B)**. **(C, D)** Case 2: complete villous atrophy with intraepithelial lymphocytosis (>25%) before vedolizumab treatment **(C)**, and subtotal villous atrophy (Marsh IIIb) after treatment **(D)**. **(E, F)** Case 4: complete villous atrophy with intraepithelial lymphocytosis (>30%) before vedolizumab treatment **(E)** and normal villous architecture in the most recent biopsy obtained after treatment **(F)**.

## Discussion

In this case series, three of the four patients treated with vedolizumab achieved an initial clinical response. All three patients with complete villous atrophy experienced initial clinical improvement, and one of them (Case 1) also showed improvement in ileal and colonic involvement. Among the patients who initially responded, Case 2 had only mild histological improvement despite the initial clinical response, and experienced recurrence of symptoms after two years of treatment. In contrast, the patient with severe, refractory pancolitis (Case 3) showed no clinical or histological improvement.

Inflammatory enteropathy occurs between 5% to 21% of patients with CVID across large cohorts, and appears to be associated with the subtype of patients found to have defective T cell function (9–11). Three out of four patients had very low B cells counts, and all of them had decreased absolute CD4 T cells. Previous reports have focused on patients with CVID, whereas we also describe the response to vedolizumab in patients with combined immunodeficiency, who showed a good initial clinical response to treatment, although one of them experienced mild histologic response and recrudescence of symptoms after two years of therapy. It is important to note that, at the time vedolizumab was initiated, none of these patients had a confirmed molecular diagnosis. The decision was based on the unclear underlying immune pathway, a history of recurrent or severe infections, and the preference for a selective agent with a more favorable infectious safety profile.

Previous reports of vedolizumab in immunodeficiency-associated enteropathy have yielded heterogeneous results. Boland et al. and Akhtar et al. reported clinical, endoscopic, and histologic improvement in patients with CVID-associated enteropathy, consistent with our findings among patients with predominant villous atrophy (7, 8). In contrast, Sifers et al. described acute clinical deterioration during vedolizumab induction in a subset of patients with CVID, including two patients with concomitant infections (5). Additionaly, Deng et al. reported initial gastrointestinal improvement followed by the development or worsening of extraintestinal inflammation in two patients with CVID or XLA (12). These observations suggest that the inflammatory mechanisms underlying immunodeficiency-associated enteropathy are heterogeneous and may not uniformly depend on α4β7-mediated lymphocyte trafficking, as well as its gut-selective mechanism is insufficient in patients with prominent systemic inflammation.

Several lines of evidence from IBD may help explain this heterogeneity. Clinical and biological predictors of vedolizumab response remain incompletely defined, but greater disease activity, higher inflammatory burden, and previous anti-TNF exposure have generally been associated with lower rates of response (13). Importantly, mucosal immunological features may be more informative than clinical disease severity alone. Rath et al. demonstrated that vedolizumab responders had higher baseline intestinal α4β7 expression, whereas non-remitters showed a tissue-destructive, leukocyte-driven inflammatory profile characterized by activation of TNF-dependent pathways. In contrast, responders exhibited a comparatively less inflammatory transcriptional profile, with predicted inhibition of TNF and IFN-γ signaling (14). Subsequent transcriptomic studies similarly identified dysregulation of TNF receptor/NF-κB, interleukin, MyD88, and Toll-like receptor pathways among vedolizumab nonresponders (15). Although these biomarkers were not assessed in our patients, the refractory pancolitis observed in Case 3 may reflect a more established and tissue-destructive inflammatory phenotype in which blockade of α4β7-dependent trafficking alone is insufficient.

In patients with inborn errors of immunity, additional disease-specific factors may further influence treatment response. Sifers et al. found no significant difference in peripheral α4β7+ T-cell frequencies between CVID patients with and without gastrointestinal disease, suggesting that the mere presence of circulating α4β7+ lymphocytes may not capture the relevant pathogenic mechanism (5). Moreover, concomitant viral or bacterial infections may independently drive gastrointestinal inflammation and clinical deterioration, potentially mimicking or contributing to treatment failure. Genetic heterogeneity may represent another source of variability: in the Sifers series, a patient with a CTLA4 mutation improved with vedolizumab, whereas patients with BACH2 and NFKB1 variants experienced adverse clinical courses (5). Vedolizumab has also shown efficacy in immune checkpoint inhibitor-induced enterocolitis, including colitis induced by CTLA-4 blockade with ipilimumab. Both pharmacologic CTLA-4 blockade and CTLA4 haploinsufficiency can lead to dysregulated T-cell activation and intestinal inflammation, which support the concept that vedolizumab may be most effective when intestinal pathology remains substantially dependent on aberrant lymphocyte trafficking. (16). Interestingly, TRAF3 haploinsufficiency has also been reported in a pediatric patient with very-early-onset IBD who, in contrast to our Case 1, presented with elevated immunoglobulin levels and expanded T-follicular helper cells and achieved remission with ustekinumab after failing azathioprine and infliximab (17). Our patient, in contrast, had hypogammaglobulinemia and achieved a sustained intestinal response to vedolizumab. This immunological divergence suggests that TRAF3 haploinsufficiency may give rise to heterogeneous immune phenotypes in which different inflammatory pathways predominate. Regarding Case 4, although she is the only patient who received rituximab, it is worth noting that her gastrointestinal disease developed before rituximab administration, and that gastrointestinal remission was not accompanied by humoral reconstitution, as agammaglobulinemia persists. These observations are hypothesis-generating rather than evidence of genotype-specific efficacy, but they underscore the biological heterogeneity of enteropathy associated with IEI.

Two patients had infections during treatment with vedolizumab, both of them affecting the gastrointestinal tract (CMV, norovirus and *Clostridium difficile*). None of them had systemic infections during treatment. These gastrointestinal infectious complications may represent either a trigger of uncontrolled intestinal inflammation or, alternatively, a consequence of treatment itself. Concerning the risk of severe CMV infection, it has been suggested either as primary infection or reactivation while under vedolizumab treatment, although this is based on case reports in patients with IBD (18). Conversely, other reports describe resolution of CMV infection under vedolizumab treatment (19, 20). Notably, in one of those cases, despite the resolution of CMV infection under vedolizumab, the patient developed recurrent symptoms after several months of treatment and ultimately required surgery (19). Regarding norovirus, there is a wide spectrum of manifestations including villous atrophy and malabsorption (21). It is postulated that norovirus infection may either exacerbate an underlying inflammatory enteropathy or itself accounts for symptom recrudescence, contributing to inflammatory changes that persisted despite vedolizumab therapy, and the two possibilities are being considered in the Case 2. Regarding *Clostridium difficile*, extensive evidence from patients with IBD indicates that vedolizumab was not associated with an increased risk of *Clostridium difficile* infection (22, 23). However, data on its safety in patients with IEI remain lacking. Finally, a previous case series of patients with CVID reported acute clinical deterioration following vedolizumab induction therapy in three patients, two of whom had concomitant infections (norovirus and *Campylobacter*) (5).

Beyond infectious complications, vedolizumab has also been linked to non-infectious adverse events: acute interstitial nephritis, described in a systematic review of nine adult cases and, more recently, in a child with very-early-onset IBD; drug-induced liver injury in a patient with ulcerative colitis and autoimmune hepatitis; and infusion-related hypersensitivity reactions, including an atypical delayed presentation with recurrent chest pain (24–27). Long-term data from the GEMINI long-term safety study showed that the most frequent adverse events over 8 years were disease exacerbation and nasopharyngitis, without new safety signals for infections, malignancy, infusion reactions, or hepatic events (28). These considerations should also be weighed against individual comorbidities when considering vedolizumab for patients with immunodeficiency-associated enteropathy.

Several practical questions remain unanswered. Among patients who respond to vedolizumab, the optimal duration of therapy and the appropriate timing for treatment discontinuation remain unknown. Our cohort provides valuable insights into the long-term effects of vedolizumab, an aspect that has been scarcely addressed in the available literature, where follow-up of patients with initial clinical response barely exceeds one year (7). Case 4 maintained clinical and endoscopic remission for several years after vedolizumab discontinuation, with an asymptomatic transient episode of mild villous atrophy that resolved after a short course of budesonide, whereas Case 1 experienced disease relapse one year after stopping therapy, highlighting the need for additional data to identify predictors of durable remission and to define the optimal duration of treatment.

On the other hand, fecal calprotectin has been proposed as a useful screening tool for gastrointestinal involvement in CVID, correlating with disease probability and reducing the need for endoscopy in low-risk patients (29). However, its role as a treatment target, rather than a screening tool, remains unestablished in this population. In our series, fecal calprotectin levels decreased in patients who responded clinically; however, discrepancies between fecal calprotectin levels and clinical and histological findings were observed during follow-up, and complete normalization was achieved in only one case. This raises the question of whether biochemical remission is a necessary therapeutic target in patients with immunodeficiency-associated enteropathy.

This study has limitations inherent to its retrospective design, small sample size, and heterogeneous endoscopic follow-up across patients, which limit the generalizability of these findings. Further studies are needed to confirm these preliminary observations and to better define the role of vedolizumab in patients with immunodeficiency-inflammatory enteropathy.

In our experience, all patients with prominent small-bowel enteropathy and villous atrophy experienced initial clinical improvement, although the response was not sustained in every case. The patient with severe refractory pancolitis showed no response. The variability in treatment response may reflect the heterogeneity of the underlying immune defects, the coexistence of gastrointestinal infections, and differences in disease severity among patients. Further studies are needed to identify predictors of response and better define which patients may benefit from this therapy. In the early stages of disease, particularly in patients in whom no molecular defect has yet been identified, vedolizumab may represent a potentially useful therapeutic option with a gut-selective mechanism to consider for immunodeficiency-associated enteropathy.

## Data Availability

The original contributions presented in the study are included in the article/supplementary material. Further inquiries can be directed to the corresponding author.
